# UV-B Stress-Triggered Amino Acid Reprogramming and ABA-Mediated Hormonal Crosstalk in *Rhododendron chrysanthum* Pall.

**DOI:** 10.3390/plants13162232

**Published:** 2024-08-12

**Authors:** Wang Yu, Xiangru Zhou, Hongwei Xu, Xiaofu Zhou

**Affiliations:** Jilin Provincial Key Laboratory of Plant Resource Science and Green Production, Jilin Normal University, Siping 136000, China

**Keywords:** UV-B, metabolomics, *R. chrysanthum*, plant hormone

## Abstract

Increased UV-B radiation due to ozone depletion adversely affects plants. This study focused on the metabolite dynamics of *Rhododendron chrysanthum* Pall. (*R. chrysanthum*) and the role of ABA in mitigating UV-B stress. Chlorophyll fluorescence metrics indicated that both JA and ABA increased UV-B resistance; however, the effect of JA was not as strong as that of ABA. Metabolomic analysis using UPLC−MS/MS (ultra-performance liquid chromatography and tandem mass spectrometry) revealed significant fluctuations in metabolites under UV-B and ABA application. UV-B decreased amino acids and increased phenolics, suggesting antioxidant defense activation. ABA treatment upregulated lipids and phenolic acids, highlighting its protective role. Multivariate analysis showed distinct metabolic clusters and pathways responding to UV-B and ABA, which impacted amino acid metabolism and hormone signal transduction. Exogenous ABA negatively regulated the JA signaling pathway in UV-B-exposed *R. chrysanthum*, as shown by KEGG enrichment. This study deepens understanding of plant stress-tolerance mechanisms and has implications for enhancing plant stress tolerance through metabolic and hormonal interventions.

## 1. Introduction

In recent years, due to the increase in greenhouse gas concentration, climate change has been further aggravated, and stratospheric ozone has been affected accordingly, leading to an increase in solar ultraviolet radiation (especially UV-B, 280–315 nm) on the Earth’s surface [1,2]. Despite being a non-biological stressor and only constituting a minor fraction of the overall ultraviolet radiation, UV-B has various negative effects on plants: plant growth rates are often decreased, photosynthesis is partially obstructed, and plant biochemistry is changed [3,4,5,6]. UV-B stress can disrupt proteins in plant photosystem II and damage electron donors, lowering plant photosynthetic rates [7,8]. Plants growing at high latitudes adapt to the effects of UV-B by altering the physiological shape of their leaves, for instance, by reducing their size and inducing them to thicken [9]. In addition, many studies have shown that UV-B leads to large fluctuations in the contents of metabolites in plants and that plants develop resistance in this way [10,11,12].

Changbai Mountain in Jilin Province of China is home to *R. chrysanthum*, which thrives at a modest height of 1300 to 2650 m [13]. The harsh climate and soil conditions at the summit of Changbai Mountain present a formidable obstacle to vegetation flourishing in this area. Consequently, the capacity of *R. chrysanthum* to withstand abiotic stress has been consistently improved throughout the extensive process of evolution [14,15,16].

Abscisic acid, an important phytohormone, plays an important role in seed rest, stomatal regulation, and gene expression [17,18]. It is also a key signaling molecule for plants against abiotic stress factors. Indeed, plant responses to abiotic stresses and the mobilization of their own defense mechanisms are largely affected by the broad and deep influence of ABA [19]. ABA can regulate plant growth, leaf color, and growth rate, thus mitigating the effects of abiotic stress on plants. Not only that, ABA can also regulate plant abiotic stress responses by regulating phytohormone levels, influencing enzyme activity and altering the way in which plant cell membranes are transported [20,21]. Following a thorough proteomic analysis, it was discovered that exogenous ABA might greatly increase rice’s tolerance to abiotic stressors, giving it a higher chance of surviving hardship [22]. Exogenous ABA also boosts a plant’s cold tolerance by increasing its soluble sugar and proline contents, enabling it to thrive in extremely cold temperatures [23,24]. An earlier study’s thorough analysis of phosphorylation transcriptomics revealed that exogenous ABA might activate *R. chrysanthum’*s downstream transcription factors. *R. chrysanthum’*s stomatal closure was brought on by this activation mechanism, which significantly increased the plant’s ability to withstand stress [25]. Nevertheless, there are not many studies on how exogenous abscisic acid increases *R. chrysanthum’*s UV-B resistance, such that it is worth delving further into its resistance mechanism.

Several studies have found that JA (jasmonic acid) is vital in plant responses to various stressors. When grapes are subjected to drought stress, JA improves their drought tolerance capability [26,27,28]. Furthermore, JA derivatives can actively help plants cope with a variety of harsh environmental situations [29]. Exogenous MeJA has been demonstrated to influence the flavonoid-related pathway in Begonia, allowing it to respond positively to ozone stress [30]. Nevertheless, plant hormones interact in intricate ways, and other hormones regulate JA [31,32]. The connection between the roles of ABA and JA in *R. chrysanthum* during UV-B stress has not yet been investigated. Given the critical functions of ABA and JA, this study combined chlorophyll fluorescence indexes and metabolomic data to investigate the intricate regulatory interactions among hormones in *R. chrysanthum* in response to UV-B.

Plants possess the capacity to maintain their regular development and procreation and can withstand diverse pressures as a result of the interplay of multiple metabolites generated within the plant. Metabolomics has become a reliable tool for investigating how plants can withstand different pressures at the metabolite level as its technology has advanced over time [33,34]. Plants can alter the effects of UV-B on themselves through their own internal metabolites and even other abiotic or biological interactions. Based on the qualitative and quantitative analysis of metabolites, metabolomics can be used to analyze metabolic pathways or metabolic networks and better reveal the unique coping mechanisms developed by plants in response to various adverse environmental factors. In this experiment, the metabolites of *R. chrysanthum* treated with UV-B as well as ABA were analyzed multivarially by ultra-performance liquid chromatography and tandem mass spectrometry (UPLC-MS/MS). These results will help to study the complex process of plant acclimatation to UV-B.

Therefore, based on the results obtained by previous authors that exogenous ABA can enhance the UV-B stress resistance of *R. chrysanthum* [35], the present experiment was conducted to further investigate the molecular mechanism of *R. chrysanthum*’s response to UV-B stress and the effects exerted by exogenous ABA through a metabolomic perspective. Furthermore, the regulatory roles of JA and ABA in *R. chrysanthum* following UV-B exposure were disclosed by integrating chlorophyll fluorescence characteristics with metabolomic data, elucidating the intricate molecular mechanisms of its numerous hormones in response to UV-B.

## 2. Result

### 2.1. Exogenous Hormone Therapy Improves R. chrysanthum’s Ability to Withstand UV-B Damage

To investigate the damaging impact of UV-B radiation on *R. chrysanthum* and the role of exogenous hormone treatments in enhancing its resistance to UV-B, chlorophyll fluorescence characteristics were measured for the experimental materials following various experimental treatments.

**Figure 1:**
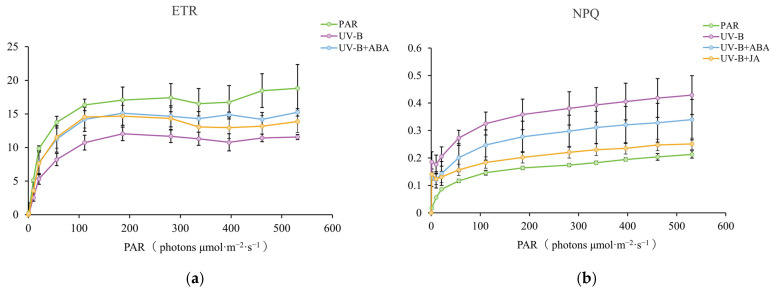

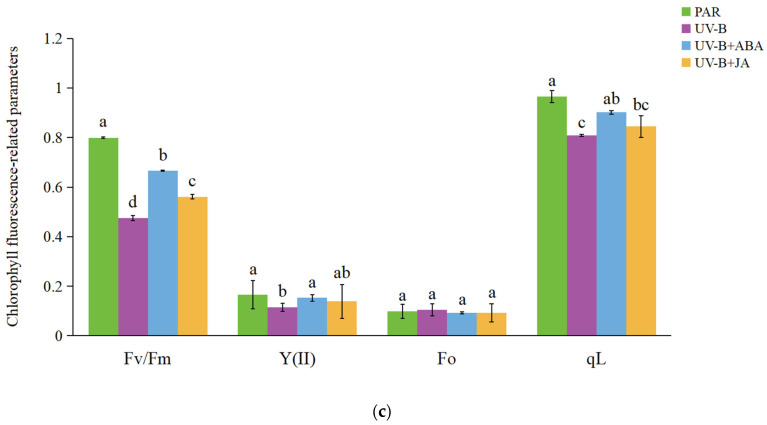
The chlorophyll fluorescence characteristics of the experimental materials altered in accordance with UV-B light and exogenous hormones (100 μmol/L). (**a**,**b**) ETR and NPQ foldplots of *R. chrysanthum*. The PAR (photosynthetic active radiation) treatment group was used as a negative control to show the status of chlorophyll fluorescence parameters of *R. chrysanthum* in the absence of UV-B radiation. Data were analyzed by Origin 2021. (**c**) Histogram of various photosynthetic parameters of *R. chrysanthum*. The heights of the bars depict the means of three biological duplicate experiments (*n* = 3), while the error bars depict the standard deviations of the samples. Different letter markers indicate significant differences across data groups (*p* < 0.05).

The findings revealed that UV-B radiation significantly reduced *R. chrysanthum’*s ETR (actual electron transport rate), whereas exogenous hormone therapies (ABA and JA) significantly reversed this tendency and raised the ETR (Figure 1a). NPQ (non-photochemical quenching) is a photoprotective process in plants that dissipates excess light energy absorbed during photosynthesis as heat, protecting the photosynthetic machinery from photodamage. The results of this study showed a significant increase in NPQ after UVB radiation compared to PAR-treated controls. Following exogenous hormones, NPQ no longer increased, implying that both hormones helped *R. chrysanthum* disperse surplus excitation energy, allowing it to respond positively to UV-B light (Figure 1b).

Fv/Fm (maximal photochemical efficiency of PSII) and Y (II) (photochemical yield of PSII) reflect PS II’s maximal and actual photochemical efficiencies, respectively. Fo (minimal fluorescence) is the plant’s initial fluorescence, which typically increases when it is stressed. qL can indicate plant photosynthetic activity. Relative to the PAR-treated group, the UV-B radiation treatment caused a significant decrease in Fv/Fm, Y(II), and qL and a non-significant increase in Fo. In contrast, exogenous ABA treatment resulted in a significant increase in Fv/Fm, Y(II), and qL and a non-significant decrease in Fo compared to the UV-B radiation-treated group. Exogenous JA treatments acted similarly to ABA treatments, resulting in a significant increase in Fv/Fm, non-significant increases in Y(II) and qL, and a non-significant decrease in Fo, as compared to the UV-B radiation-treated group (Figure 1c).

Overall, the discoveries demonstrated that UV-B radiation destroyed the photosynthetic systems of the experimental materials, while exogenous hormone therapies protected them. This demonstrates that ABA and JA play significant parts in *R. chrysanthum’*s reactions to UV-B stress and that there may be a regulatory link between the two hormones under UV-B stress. According to the outcomes, while these two hormones showed some protective effects on *R. chrysanthum*, ABA was more effective in increasing *R. chrysanthum’*s resistance than JA.

### 2.2. Negative Control of JA by ABA in R. chrysanthum during UV-B Stress

To investigate the interactions between *R. chrysanthum*’s two hormones (ABA and JA) under UV-B radiation, the contents of JA and its derivative JA-Val (Jasmonoyl-L-valine) were determined in this experiment utilizing widely targeted metabolomics after therapy with UV-B radiation and exogenous ABA.

**Figure 2:**
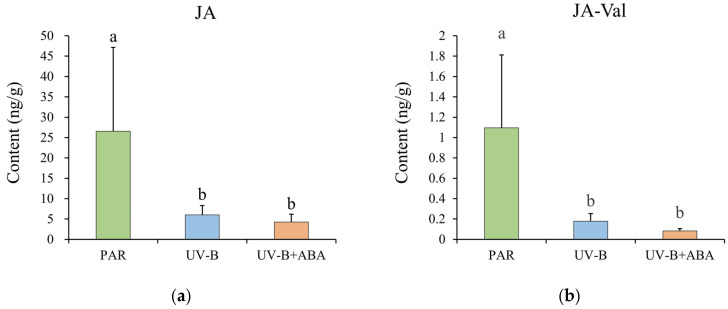
Exogenous ABA altered the levels of JA and its products in UV-B-treated experimental materials. (**a**) Histogram of JA content. (**b**) Histogram of JA-Val content. The heights of the bars depict the means of three biological duplicate experiments (*n* = 3), while the error bars depict the standard deviations of the samples. Different letter marks indicate significant differences across data groups (*p* < 0.05).

The results showed that UV-B significantly reduced the contents of JA and JA-Val in *R. chrysanthum* compared to the control group treated with PAR. While exogenous ABA treatment did not significantly alter the JA and JA-Val contents of *R. chrysanthum* exposed to UV-B radiation compared to the UV-B treatment group, they were nonetheless reduced (Figure 2a,b). This shows that ABA may have an inhibitory effect on JA in *R. chrysanthum* when exposed to UV-B light. This is also consistent with the results for chlorophyll fluorescence parameters. Although exogenous JA treatment enhanced the radiation resistance of *R. chrysanthum*, the effect was not as obvious as that of exogenous ABA treatment, and it is possible that the exogenous JA was regulated by *R. chrysanthum*’s own ABA.

### 2.3. Metabolomic Analysis of R. chrysanthum

**Figure 3:**
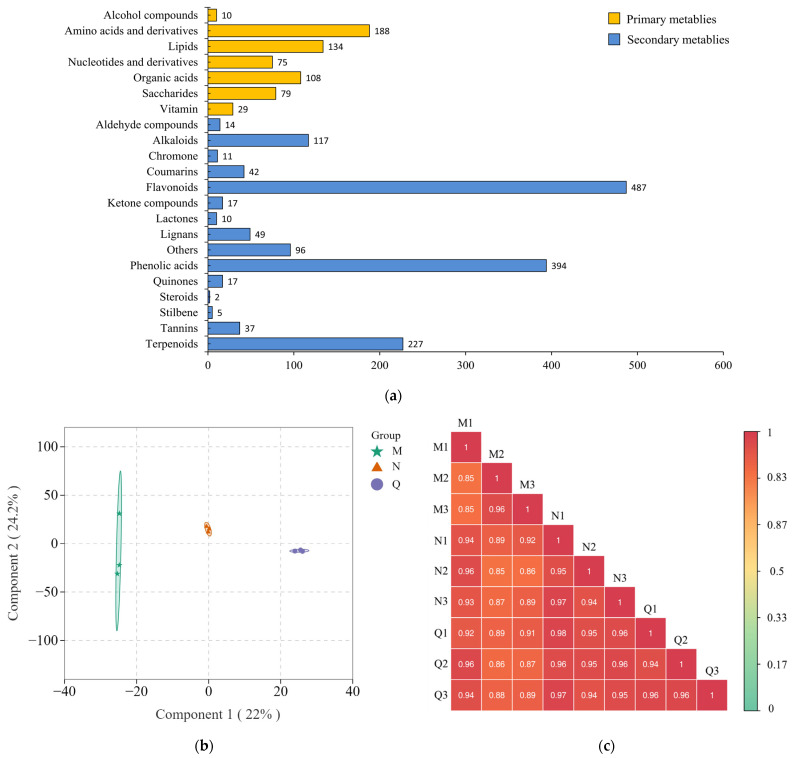
Metabolomic analysis of *R. chrysanthum* under UV-B stress. (**a**) Statistics on the quantities of different types of primary and secondary metabolites. Data were analyzed by Origin 2021. (**b**) Orthogonal partial least-squares discriminant analysis (OPLS-DA) of each sample after UV-B radiation treatment. OPLS-DA was centered after log2 transformation of the raw data. Analyses were performed using the MetaboAnalystR package OPLSR and the Anal function in R software (1.0.1). (**c**) Pearson’s correlation coefficients (PCCs) between the quality control samples (QCs) and each sample. The Pearson’s correlation coefficients were calculated using the inbuilt cor function in R software.

To determine the reaction of *R. chrysanthum* to UV-B stress and the function of exogenous ABA in this regard, a UPLC-MS platform was used to extensively target metabolomic technology to identify primary and secondary metabolites in the samples. A total of 2148 metabolites (623 primary metabolites and 1525 secondary metabolites) were detected, including 188 amino acids and derivatives, 75 nucleotides and derivatives, 134 lipids, 487 flavonoids, 49 lignans, 42 coumarins, 37 tannins, 117 alkaloids, 394 phenolic acids, 227 terpenoids, 108 organic acids, 2 steroids, 5 stilbenes, 17 ketone compounds, 11 chromones, 14 aldehyde compounds, 10 lactones, 10 alcohol components, 17 quinones, 79 saccharides, 29 vitamins, and 96 others (Figure 3a; Supplementary Table S1). Thus, amino acids and derivatives, organic acids, and lipids are the major primary metabolites of *R. chrysanthum*, while flavonoids, phenolic acids, and terpenoids are the major secondary metabolites of *R. chrysanthum*.

By conducting OPLS-DA (orthogonal partial least-squares discriminant analysis) on samples, differences between different groups can be observed. Therefore, in this experiment, three independent replicates of PAR-treated (M), UV-B radiation-treated (N), and exogenous ABA-treated (Q) samples of *R. chrysanthum* were further analyzed for metabolites using OPLS-DA (Figure 3b). The outcomes demonstrated that the first principal component could explain 22% of the features of the original dataset, while the second principal component could explain 24% of the features of the original dataset. This suggested that after exogenous ABA treatment and UV-B radiation treatment, there were notable metabolite alterations in the samples. At the same time, Pearson correlation analysis was conducted on the samples, including quality control samples (QCs), and the results showed a high positive correlation between each duplicate sample (Figure 3c). This indicates better biological replication for each of the three comparison groups (M, N, and Q). In summary, the relevant data from this experiment were reliable and suitable for subsequent analysis.

### 2.4. Response of Primary and Secondary Metabolites of R. chrysanthum to UV-B as Well as Exogenous Exogenous ABA

**Figure 4:**
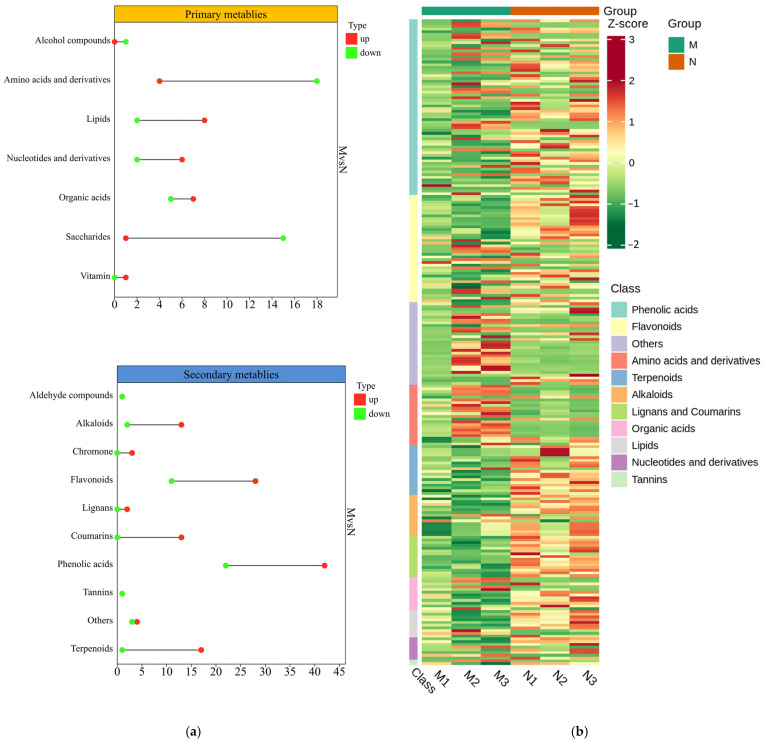

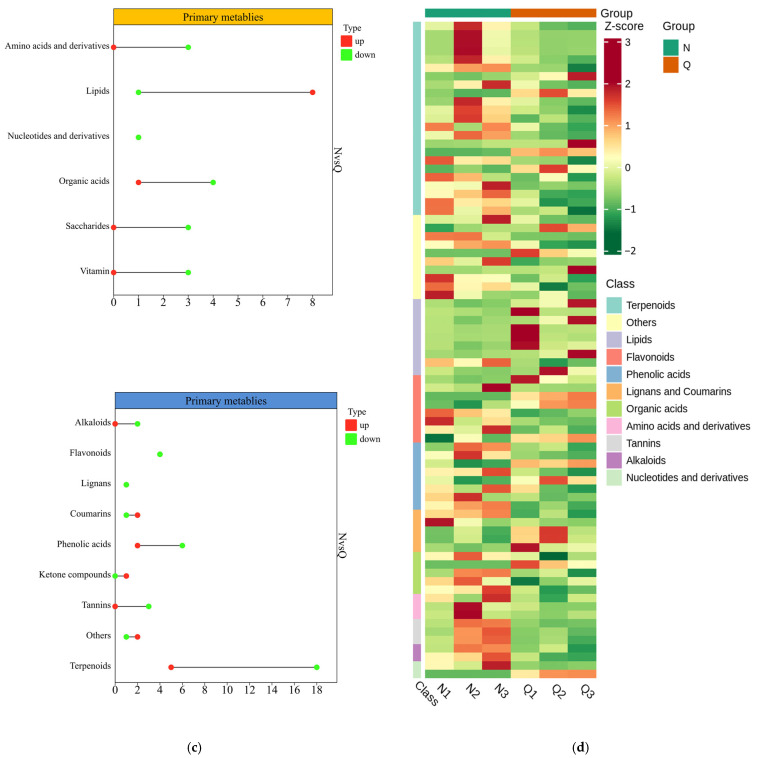
Analysis of DMs in *R. chrysanthum* under UV-B radiation and exogenous ABA treatment. (**a**) Statistics on the upregulation and downregulation of primary and secondary differential metabolites produced by UV-B treatment. Plotted by chiplot (www.chiplot.online (accessed on 21 September 2023)), a free online data analysis website. (**b**) Heatmap of clustering composed of differential metabolites produced by UV-B treatment. The horizontal coordinate is the name of the sample, the vertical coordinate is the first-level classification of the differential metabolite, the different colors represent the different values obtained from the standardization of the different relative contents (red for high content, green for low content), “Group” refers to the grouping, and “Class” refers to the first-level classification of the substance. The clustered heatmaps applied UV (unit variance scaling) processing for the raw relative contents of the differential metabolites by rows, and these were graphically plotted via Metware Cloud (https://cloud.metware.cn (accessed on 23 October 2023)), a free online data analysis platform. (**c**) Statistics on the upregulation and downregulation of primary and secondary differential metabolites produced by exogenous ABA treatment. (**d**) Heatmap of clustering composed of differential metabolites produced by ABA treatment.

The DMs (differential metabolites) of *R. chrysanthum* treated with UV-B and exogenous ABA were visualized using heatmaps, and quantitative statistics on the upregulation and downregulation of related primary and secondary metabolites were visualized through dumbbell plots. The dumbbell plots showed that after UV-B radiation, a total of 70 primary DMs and 165 secondary DMs were generated (Figure 4a; Supplementary Table S2), while after exogenous ABA treatment, 25 primary DMs and 53 secondary DMs were generated (Figure 4c; Supplementary Table S3). The cluster heatmap showed that the contents of some primary and secondary metabolites in *R. chrysanthum* fluctuated after UV-B radiation treatment (Figure 4b). The primary metabolites that were mainly upregulated included lipids, nucleotides and their derivatives, and organic acids. The contents of flavonoids, terpenoids, and phenolic acids among secondary metabolites increased, while the content of saccharides decreased. It is noteworthy that UV-B radiation decreased the contents of most amino acids and derivatives in *R. chrysanthum* but stimulated an increase in phenolic contents. After the application of exogenous ABA, lipids dominated the upregulated primary metabolites, indicating their involvement in responding to ABA resistance to UV-B stress. However, the terpenoids in secondary metabolites decreased significantly (Figure 4d).

### 2.5. Primary and Secondary Main Contributing Metabolites

**Figure 5:**
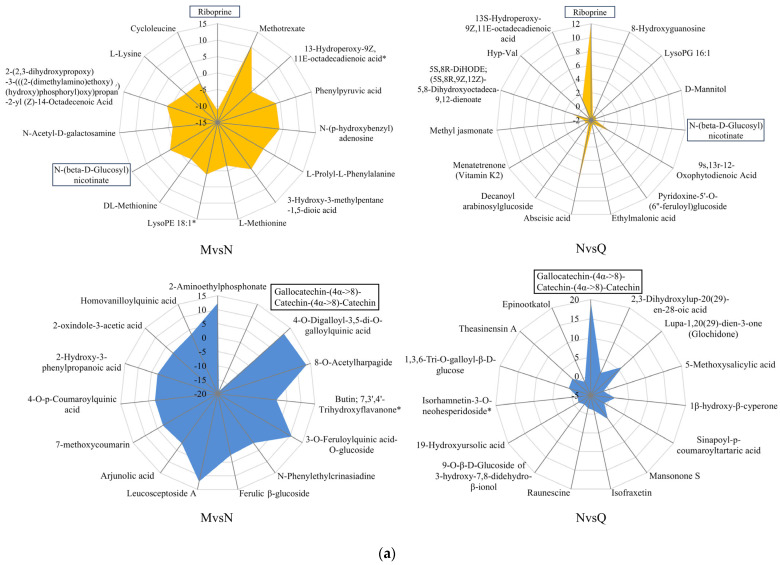

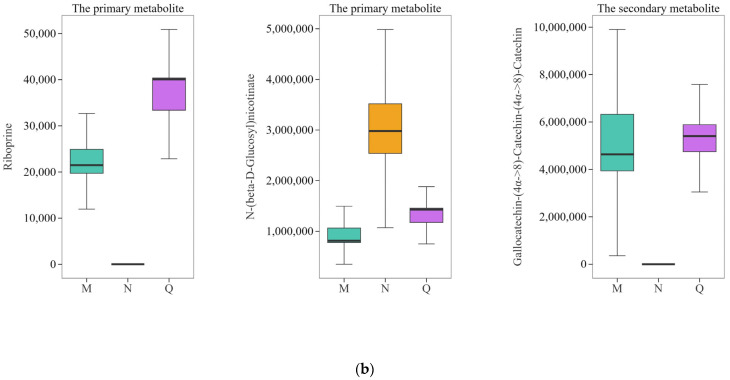
Multivariate analysis of DMs in *R. chrysanthum* under UV-B stress and exogenous ABA. (**a**) The radar image shows the top 15 DMs screened based on VIP values under UV-B stress and exogenous ABA treatment. Data were analyzed by Origin 2021. (**b**) Primary and secondary main contributing metabolites. The horizontal lines at the top and bottom of the box plots represent the maximum and minimum values, respectively, and the horizontal lines inside the boxes represent the median. Graphing by Metware Cloud (https://cloud.metware.cn (accessed on 18 October 2023)), a free online data analysis platform.

To further explore which primary and secondary metabolites respond to UV-B stress and exogenous ABA treatment, a radar map based on variable importance in projection (VIP) was used to compare and screen the primary and secondary metabolites under the two treatments (Figure 5a). The results showed that three metabolites—riboprine, N-(beta-D-glucosyl)nicotinate, and gallocatechin-(4α->8)-catechin-(4α->8)-catechin—were the major contributing metabolite groups, implying that they are functionally related to the UV-B radiation response. Among them, riboprine and gallocatechin-(4α->8)-catechin-(4α->8)-catechin showed similar trends after UV-B radiation as well as exogenous ABA treatment, with a significant decrease in the contents of both of them after UV-B radiation and an increase in the contents of both of them after the application of exogenous ABA. UV-B radiation increased the content of N-(beta-D-glucosyl)nicotinate, yet it was not further elevated by exogenous ABA (Figure 5b).

### 2.6. Effects of UV-B and Exogenous ABA Treatment on the Metabolic Pathway of R. chrysanthum

All DMs in the different comparison groups (MN and NQ) were matched to KEGG entries to obtain pathway information involving metabolites. Enrichment analysis of the annotated results was conducted to obtain pathways with more enriched DMs.

**Figure 6:**
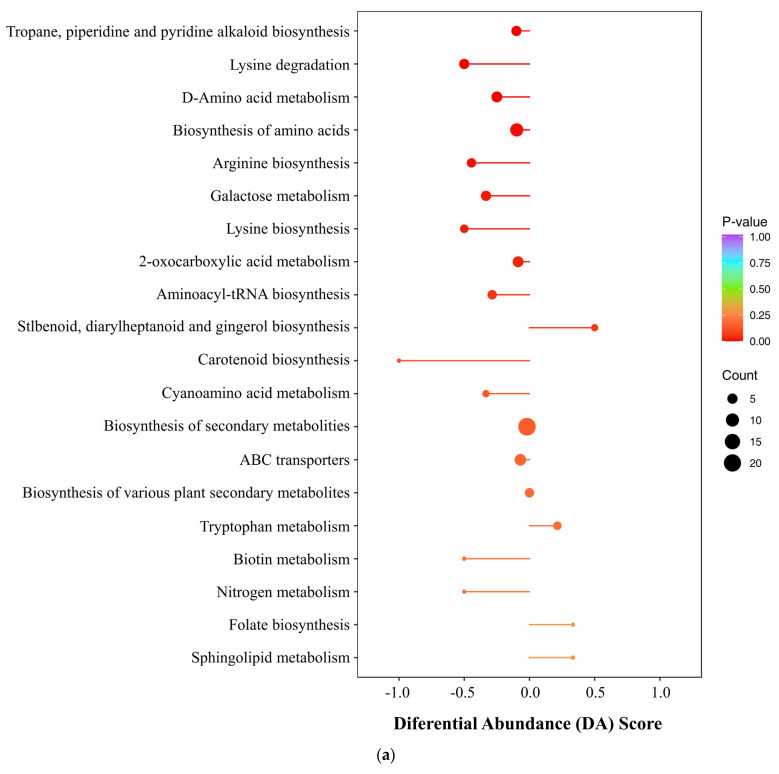

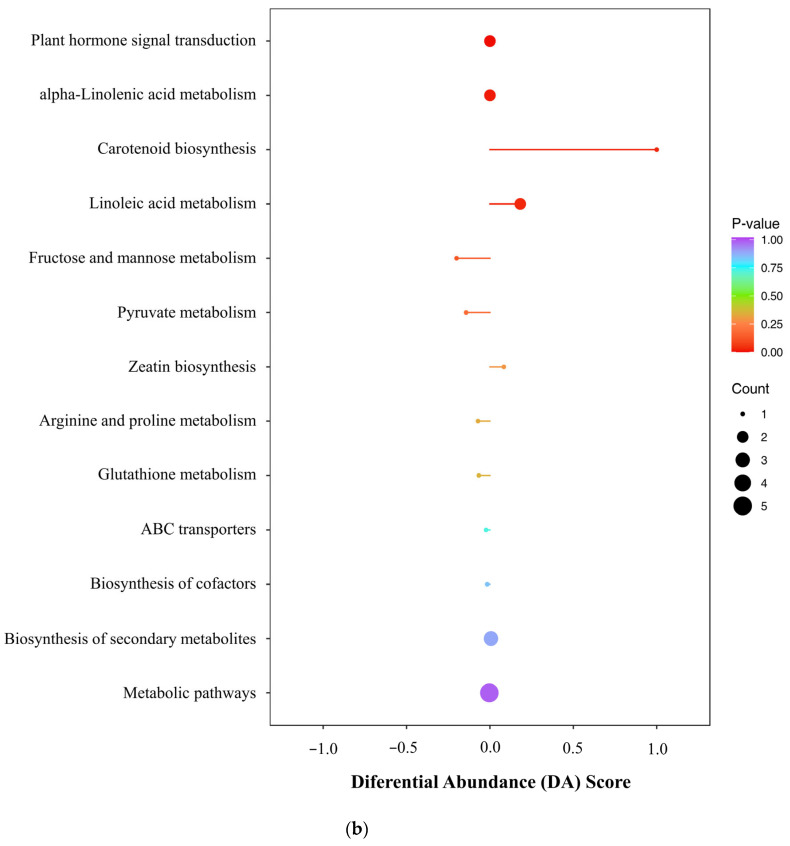
(**a**) Differential abundance (DA) of DMs treated by UV-B. (**b**) Differential abundance (DA) of DMs treated by ABA. The horizontal coordinates represent the differential abundance scores, which were calculated as the ratio of the difference between upregulated and downregulated metabolites involved in the pathway to the number of all metabolites involved in the pathway. The length of the line segments represents the absolute value of the DA score, the size of the dot at the end of the line segments represents the number of differential metabolites in the pathway, and the color of the line segments and dots reflects the *p*-value size (the closer it is to red, the smaller the *p*-value, and the closer it is to purple, the larger the *p*-value). Graphing by Metware Cloud (https://cloud.metware.cn (accessed on 6 May 2023)), a free online data analysis platform.

The DMs of Group M and Group N were mainly annotated and enriched in amino acid metabolism-related pathways. These results suggest that UV-B radiation caused significant changes in several amino acid-related metabolic pathways in *R. chrysanthum*. This indicates that *R. chrysanthum* likely primarily adapts to UV-B radiation by reprogramming pathways related to amino acid metabolism, e.g., lysine degradation, D-amino acid metabolism, arginine biosynthesis, lysine biosynthesis, and other pathways (Figure 6a).

The DMs of Group N and Group Q were mainly annotated and enriched in plant hormone signal transduction, alpha-linolenic acid metabolism, and linoleic acid metabolism. Alpha-linolenic acid metabolism is involved in JA synthesis, and plant hormone signal transduction is also related to JA signaling, indicating that ABA in *R. chrysanthum* following UV-B stress does indeed exert a regulatory effect on JA.

In these comparative groups (MN and NQ), some metabolic pathways overlapped, including carotenoid biosynthesis, ABC transporters, and biosynthesis of secondary metabolites. Exogenous ABA promoted linoleic acid metabolism, suggesting that ABA enables *R. chrysanthum* to produce more phytolipids with energy-accumulating effects to mitigate the damage caused by UV-B to *R. chrysanthum*. Notably, UV-B radiation severely inhibited carotenoid biosynthesis, and exogenous ABA treatment alleviated this inhibition, suggesting that ABA may help *R. chrysanthum* to repair its UVB-damaged phototropic capacity (Figure 6b). This discovery fits nicely with the photosynthesis index data, indicating that ABA protects *R. chrysanthum*’s photosynthetic system.

### 2.7. UV-B and Exogenous ABA Prompt Rearrangement of the Metabolic Pathway Network of R. chrysanthum

**Figure 7:**
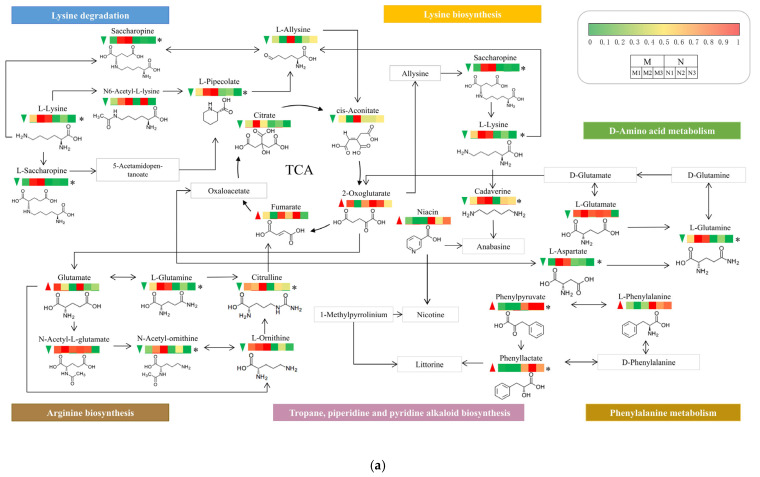

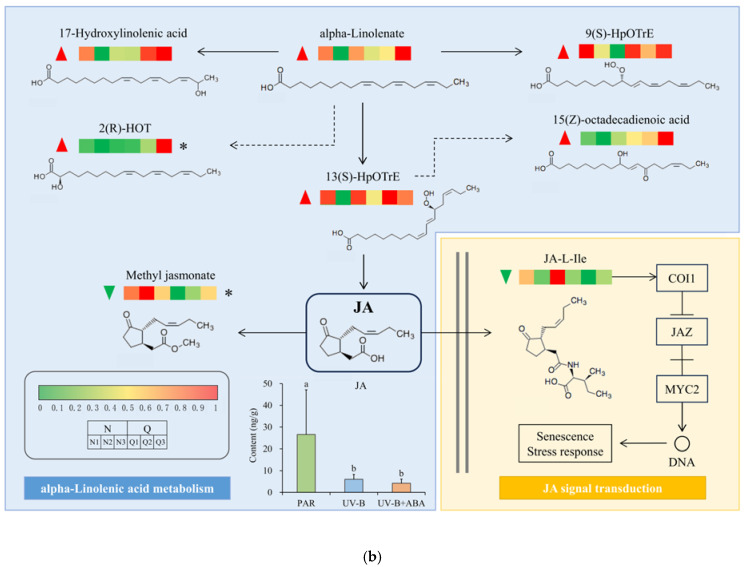
UV-B-induced metabolic pathway rearrangement network in *R. Chrysanthum*. (**a**) Simplified modeling of amino acid-related metabolic pathways exposed to UV-B radiation. The contents of the involved metabolites are presented as heatmaps after data normalization, where the red and green arrows on the left side of the heatmaps positively represent increases and decreases in metabolite contents after UV-B radiation, respectively, and “*” indicates that the relevant metabolites changed significantly. The three adjacent squares in the left half of the heatmaps represent the expression of each metabolite from three replicate experiments (*n* = 3) in group M, and the three adjacent squares in the right half of the heatmaps represent the expression of each metabolite from three replicate experiments in group N. (**b**) Simplified modeling of JA production and signaling pathways following exogenous ABA treatment. The three adjacent squares in the left half of the heatmaps represent the expression of each metabolite from three replicate experiments (*n* = 3) in group N, and the three adjacent squares in the right half of the heatmaps represent the expression of each metabolite from three replicate experiments in group Q. The heights of the bars in the graph depict the means of the three biological duplicate experiments (*n* = 3), while the error bars depict the standard deviations of the samples. Different letter markers indicate significant differences across data groups (*p* < 0.05).

The above KEGG results showed that UV-B mainly induced significant changes in several amino acid-related metabolic pathways in *R. chrysanthum*, leading to reprogramming of these pathways. However, these pathways do not exist independently but are interconnected in a network of metabolic pathways. In order to better demonstrate the interrelationships among DMs in response to radiation stress, the present experiment constructed a comprehensive metabolic network of *R. chrysanthum* after UV-B radiation based on the KEGG. The results showed that UV-B caused significant changes in many pathways in *R. chrysanthum*, such as arginine biosynthesis, lysine degradation, and D-amino acid metabolism, reflecting the fact that *R. chrysanthum* defends itself from UV-B by reprogramming these metabolic pathways. It is noteworthy that the contents of most metabolites of *R. chrysanthum* decreased after radiation, while the citric acid cycle was activated, suggesting that *R. chrysanthum* may reduce the effects of UV-B on itself by providing energy to itself through the citric acid cycle (Figure 7a).

Exogenous ABA significantly affected the alpha-linolenic acid metabolism and plant hormone signal transduction pathways in *R. chrysanthum*. Both pathways are closely related to JA, and a simplified model of these two pathways was constructed to reveal the regulatory mechanism of exogenous ABA on JA in *R. chrysanthum* under UV-B stress. The outcomes revealed that exogenous ABA increased the expression of metabolites involved in JA biosynthesis while it decreased the expression of JA derivatives (methyl jasmonate and JA-L-Ile) in *R. chrysanthum* following UV-B stress. Among them, JA-L-Ile (jasmonoyl-L-isoleucine) is a key substance in the process of JA signaling, and exogenous ABA led to a decrease in its expression. This suggests that there is indeed an inhibitory effect of ABA pairs in *R. chrysanthum* under UV-B stress in terms of JA exerting its own function (Figure 7b).

## 3. Discussion

The Earth’s environment has been deteriorating in recent years, leading to a depletion of the stratospheric ozone layer and an influx of UV-B radiation that would have otherwise been blocked, resulting in an excessive amount of radiation reaching the Earth [36]. Conversely, UV-B hinders the regular functioning of plant photosystems, hampers their transpiration, and can cause chaos throughout their entire lifespan [37].

The impacts of adversity stress on the plant photosynthetic system can be accurately represented by several chlorophyll fluorescence metrics [38,39,40]. In this case, the chlorophyll fluorescence characteristics of *R. chrysanthum* were measured, and the outcomes revealed that UV-B had a variety of detrimental effects on the experimental materials’ photosynthetic systems. After administering exogenous hormones (ABA and JA), the test materials’ UV-B resistance increased, with ABA having a stronger effect than JA (Figure 1). Exogenous ABA treatment reduced the concentration of JA and its product JA-Val in the test sample following UV-B exposure (Figure 2). While JA and ABA equally improved the stress tolerance of the experimental materials, the findings suggest that ABA may have a negative regulatory effect on JA during UV-B exposure.

In the present study, the effect of UV-B on *R. chrysanthum* as determined from a metabolomic perspective revealed large fluctuations in the contents of amino acids and their derivatives, as well as saccharides, lipids, phenolic acids, flavonoids, and terpenoids. However, upon application of exogenous ABA, it was found that lipids and terpenoids synergized with ABA to counteract the damage produced by UV-B. In addition, riboprine, N-(beta-D-glucosyl)nicotinate, and gallocatechin-(4alpha->8)-catechin-(4alpha->8)-catechin were the major contributing metabolite groups with large macro-responses after UV-B and exogenous ABA.

*R. chrysanthum* undergoes a range of adaptive processes to mitigate the detrimental effects of radiation, with the accumulation of saccharides being a crucial factor [41,42]. Indeed, there is a strong correlation between UV-B radiation and the presence of saccharides in plants. Scientific evidence has demonstrated that the presence of UV-B leads to a reduction in the overall carbon sequestration within plants [43,44]. This would indirectly impact the transportation of saccharides and the accumulation of starch, as the regulation of this process could be influenced by the carbon fluxes generated during sucrose synthesis [45]. While sucrose assumes a vital function as a saccharide in plant leaves, it also engages in various metabolic processes within plants as a means of signaling [46]. And the strength of the plant’s ability to pit against adversity is also regulated by the amount of sucrose present in the vesicles. Furthermore, the decomposition of starch, a saccharide, bestows upon plants the capacity to sustain their natural carbohydrate levels irrespective of their surroundings [47]. The results of the present study showed that UV-B radiation was indeed able to induce a decrease in the saccharide contents of most species (Figure 4c). It is therefore likely that *R. chrysanthum* plants respond to the radiation they encounter by synthesizing and consuming starch in this manner—a result that is consistent with the findings of previous studies [47].

During the daytime, plants provide themselves with energy for growth and development through photosynthesis; however, in the absence of light, plants mobilize their own metabolites, such as carbohydrates and amino acids, to provide themselves with energy through the TCA cycle and associated amino acid metabolism [48,49]. Plants are subjected to abiotic stresses that severely interfere with plant photosynthesis and lead to associated signal transduction that promotes reprogramming of plant metabolic networks [50]. The results of KEGG enrichment analyses in this experiment showed that UV-B radiation caused significant changes in several amino acid-related metabolic pathways of *R. chrysanthum*, contributing to the reprogramming of amino acid metabolic pathways (Figure 6a). Among the amino acids that were detected, an increase was observed in the content of glutamate, which has a crucial role as a core amino acid in the amino acid metabolism of higher plants and is able to participate in the synthesis of chlorophyll [51]. This suggests that *R. chrysanthum* is likely able to restore damaged chlorophyll through elevated glutamate contents and thus actively resist UV-B stress (Figure 7a). UV-B radiation similarly elevated phenylalanine contents in *R. chrysanthum*. Phenylalanine plays an important role in the synthesis of many antioxidant secondary metabolites [52]. It is likely that *R. chrysanthum* initiates its own antioxidant defense mechanisms by increasing its own phenylalanine content—a phenomenon that is also consistent with the upregulation of phenols and flavonoids that was observed after exposure to UV-B radiation (Figure 4a,b). In addition, the content of niacin (nicotinic acid) in *R. chrysanthum* increased after UV-B radiation. Niacin has the effect of repairing DNA and promotes the synthesis of ATP [53]. The experimental results suggest that *R. chrysanthum* actively responds to the damage caused by UV-B by enhancing these specific amino acids that play a role in resisting abiotic stresses. A crucial modulator of the interactions between carbon and nitrogen is fumaric acid. Fumaric acid has been demonstrated to be likely to increase in the presence of UV-B radiation [54]. Notably, comparable outcomes were seen in the current study, where UV-B led to a reduction in the majority of the *R. chrysanthum*’s amino acid contents but an increase in fumaric acid, an intermediary in the tricarboxylic acid cycle. Therefore, it is possible that *R. chrysanthum* stimulates the regular course of the TCA cycle in order to provide itself with energy to fight the damage induced by UV-B.

Abscisic acid (ABA) is an important phytohormone which is involved in many physiological processes, such as growth, development, and stress resistance in higher plants, and the content and signaling of ABA in the plant body are of great significance in regulating the plant’s response to adversity as well as its growth and development [55,56]. The KEGG enrichment findings revealed that ABA treatment of UV-B-irradiated experimental materials dramatically altered alpha-linolenic acid metabolism and the plant hormone signal transduction pathway (Figure 6b). It has been shown that alpha-linoleic acid metabolism is closely related to plant responses to abiotic stresses, especially in relation to phytohormonal defense signaling pathways, such as the biosynthesis of jasmonic acid and its derivatives. Linoleic acid metabolism generates signaling molecules to initiate the expression of defense genes, influences membrane fluidity and stability to mitigate damage to cell membrane structure and function, participates in the formation of lipids, and produces derivatives with antioxidant properties in response to abiotic stresses [57,58,59,60,61]. This is congruent with the results revealed for chlorophyll fluorescence parameters, indicating that exogenous ABA enhanced the tolerance of the experimental materials’ photosynthetic system to UV-B radiation. Therefore, it is likely that ABA stimulates the production of *R. chrysanthum* lipids through linoleic acid metabolism, which in turn repairs the photosynthetic membrane system to restore the damage caused by UV-B.

Plant defense responses to various environmental stress factors are firstly controlled by relevant plant hormones. However, hormones do not regulate responses singly; different plant hormones interact with and influence each other to form a hormone regulatory network [62]. The complex regulatory network of phytohormones and their great influence on various physiological processes, such as controlling stomatal conductance, controlling chloroplastogenesis, and influencing seed dormancy, are the main reasons why phytohormones can be used as key endogenous factors to mediate plant stress responses [63,64,65]. However, few studies on the intrinsic linkages between the hormones of *R. chrysanthum* and the interactions between the hormones and metabolites are currently available. To gain a deeper understanding of defense response mechanisms, it is crucial to study the complex interactions between different phytohormones in R. chrysanthum, especially in response to damage caused by UV-B.

Exogenous ABA treatment had a substantial effect on JA production and JA signaling pathways in UV-B-treated experimental materials, according to the results of the KEGG enrichment analyses (Figure 6b). JA-L-Ile (jasmonoyl-L-isoleucine) is the active form of jasmonic acid, which is able to release transcription factors such as MYC2 by binding to the receptor protein COI1 in the plant, which in turn affects the degradation of the JAZ protein. These transcription factors further regulate the expression of downstream genes; thus, JA-Ile plays a crucial role in plant hormone signaling [66,67]. The addition of exogenous ABA reduced the expression of JA-Ile in the test materials exposed to UV-B radiation, indicating that ABA adversely controlled the JA signal transduction pathway (Figure 7b). This validates the phenomenon observed in the chlorophyll fluorescence correlation results, which show that exogenous JA is less efficient compared to ABA in enhancing *R. chrysanthum*’s UV-B resistance (Figure 1). The rationale is that, despite the fact that exogenous JA can protect *R. chrysanthum* from UV-B damage, it is also negatively regulated by its own internal ABA. Modifications in plant metabolites tend to be controlled by enzymes and genes [68,69]. It is worth noting that the expression of metabolites involved in JA biosynthesis increased despite the fact that UV-B radiation and exogenous ABA eventually resulted in a decrease in JA content. It is speculated that this happens due to genes generally responding faster to environmental stimuli than secondary metabolism, in which related enzymes and genes play a role in JA production; their method of action warrants additional exploration.

Riboprine is a nucleoside analogue in which adenosine has been modified by substitution at the 6-amino nitrogen by a delta (2)-isopentenyl group. As a plant primary metabolite, riboprine has a regulatory effect on plant growth and enables precise biological translation processes [70,71,72]. However, there are few studies related to N-(beta-D-glucosyl)nicotinate and gallocatechin-(4alpha->8)-catechin-(4alpha->8)-catechin, which are important contributors to *R. chrysanthum*’s defense against UV-B stress and are worthy of further study.

## 4. Materials and Methods

### 4.1. Material Preparation and Experimental Treatments

The approach involving UV-B radiation and exogenous plant hormones (ABA and JA) was based on previous research, with necessary modifications [73,74]. The experimental materials were obtained from their origins and transplanted to be cultivated in an intelligent artificial climate chamber capable of simulating an alpine ecology (simulated temperatures of 18 degrees Celsius during the day and 16 degrees Celsius at night).

The three comparison groups consisted of similar *R. chrysanthum* plants grown for eight months in a simulated environment with a luminous flux of 50 μmol/($m^{2}$·s). Mixed sampling was utilized to avoid the effects of individual errors (each treatment involved three biological replicates; *n* = 3). Group M was designated the control group and was irradiated with PAR only, while groups N and Q were treated with UV-B (2.3 W/m^2^) and ABA, respectively. The duration of PAR and UV-B treatment was 16 h (the irradiation was carried out twice, each time with 8 h of radiation). The radiation source for the PAR treatment was a T5 × 14W fluorescent tube (Philips, T5 × 14 W, Amsterdam, The Netherlands). In order to produce UV-B radiation for the experiment, artificial UV-B lamps with wavelengths between 280 and 320 nm (Philips, Ultraviolet-B TL 20 W/01 RS; Amsterdam, The Netherlands) were used. The irradiance of the UV-B-treated samples was measured using a light meter (TES Elec-trical Electronic Corp., Tes-1339 Light Meter Pro.; Taipei, China) and a UV intensity meter (Sentry Optron-ICS Corp., ST-513, SHH; New Taipei City, China) in accordance with the transmission function of the long-pass filter.

The materials of group M and group N were cultured in 1/4 MS medium, and the materials of experimental group Q were cultured in 1/4 MS medium with ABA (100 μmol/L). The exogenous JA-treated group followed the experimental manipulations of the Q group. The materials of the three groups were cultured for a week in their respective media before the radiation treatment described above.

**Figure 8:**
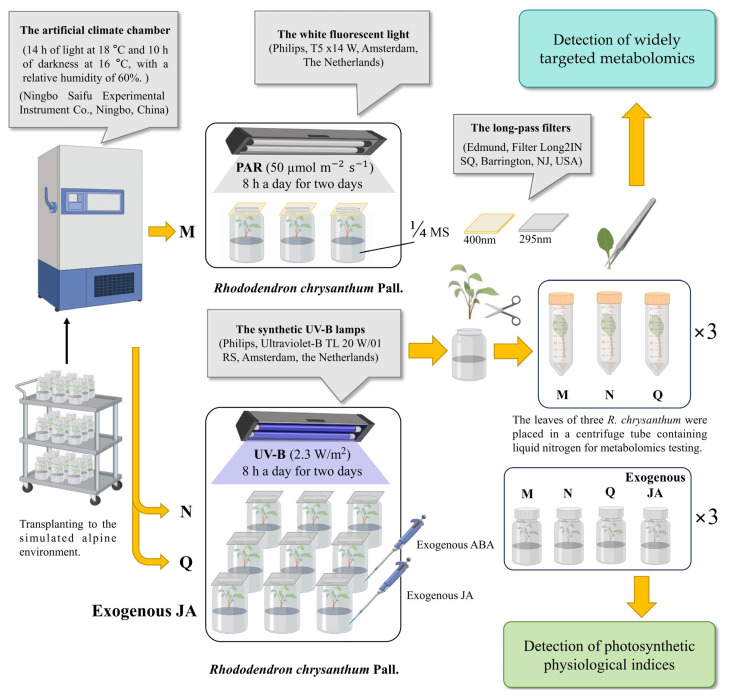
Simplified model of the experimental treatment of *R. chrysanthum*.

The *R. chrysanthum* plants exposed to UV-B radiation were covered with a 295 nm filter (Edmund, Filter Long 2IN SQ; Barrington, NJ, USA) to remove interference, and the *R. chrysanthum* plants that were not exposed to UV-B radiation were covered with a 400 nm filter (Edmund, Filter Long 2IN SQ; Barrington, NJ, USA) for radiation filtration. After the completion of each experimental treatment, subsequent multi-omics assays were performed and chlorophyll fluorescence parameters were determined (Figure 8).

### 4.2. Widely Targeted Metabolomics Testing

#### 4.2.1. Dry-Sample Extraction Conditions

The experimental methodology and the conditions for the widely targeted metabolomics were identical to the descriptions of earlier investigations [75].

Employing lyophilization via vacuum freeze-drying, the biological specimens were subjected to dehydration in a freeze-dryer (Scientz-100F, Ningbo Scientz Biotechnology Co., Ltd., Ningbo, China). Subsequently, the dehydrated samples were finely ground into a powder using a Retsch MM 400 mill (Retsch, Haan, Germany) operated at a frequency of 30 Hz for a duration of 1.5 min. An analytical balance (MS105DΜ, Mettler-Toledo International Inc., Shanghai, China) was utilized to precisely measure out 50 milligrams of the powdered sample, which was then combined with 1200 µL of a precooled (−20 degrees Celsius) 70% methanolic aqueous solution containing an internal standard. This mixture was subjected to vortexing at thirty-minute intervals for thirty seconds, and the process was repeated six times to ensure thorough extraction. Following this, the mixture was centrifuged at 12,000× *g* revolutions per minute for a period of three minutes to facilitate phase separation. The supernatant, which then contained the extracted metabolites, was carefully removed using a pipette. The resulting liquid was then passed through a 0.22 µm pore-sized microporous filter to achieve clarity, and the filtrate was subsequently transferred to an injection vial in preparation for ultra-performance liquid chromatography–tandem mass spectrometry (UPLC-MS/MS) analysis.

#### 4.2.2. UPLC Conditions

According to the previous standard procedure of Wuhan MetWare Biotechnology Co., Ltd. (Wuhan, China), a UPLC-ESI-MS/MS system (UPLC, gasket-packed UFLC CBM30A; MS, 6500 QTRAP) was used for the analysis of *R. Cruysanthum* leaf extracts [76,77]. The analysis was conducted under the following chromatographic conditions: Utilizing an UPLC system, separation was achieved using an Agilent SB-C18 column (1.8 µm, 2.1 mm × 100 mm). The mobile phase was composed of solvent A, which was pure water containing 0.1% formic acid, and solvent B, which was acetonitrile containing 0.1% formic acid. The sample analysis commenced with an initial gradient of 95% A and 5% B. Over the course of 9 min, the gradient was linearly adjusted to reach 5% A and 95% B, which was then maintained for an additional minute. Following this, the system was re-equilibrated to 95% A and 5.0% B within 1.1 min, and this composition was sustained for 2.9 min. The flow rate was maintained at 0.35 mL per minute, the column temperature was set at 40 °C, and the sample injection volume was 2 μL. The eluent was directed to an ESI-triple quadrupole-linear ion trap (QTRAP) mass spectrometer for detection.

#### 4.2.3. ESI-Q TRAP-MS/MS

The ESI source operation parameters were as follows: the source temperature was 500 °C; the ion spray voltage (IS) was 5500 V (positive ion mode) or 4500 V (negative ion mode); the ion source gas I (GSI) and gas II (GSII) and the curtain gas (CUR) were set at 50, 60, and 25 psi, respectively; and the collision-activated dissociation (CAD) was high. QQQ scans were obtained through multiple reaction monitoring (MRM) experiments, with the collision gas (nitrogen) set to medium. The declustering potential (DP) and the collision energy (CE) for each individual MRM transition were subsequently optimized. A particular set of MRM transitions was monitored for each period, in accordance with the metabolites eluted within that period.

#### 4.2.4. Qualitative and Quantitative Analysis of Metabolites

Based on the local metabolic database, the self-built MWDB (metware database; Wuhan MetWare Biotechnology Co., Ltd., Wuhan, China), and the mass spectrometry analysis, the metabolites of the samples were qualitatively and quantitatively analyzed. The characteristic ions of each substance were screened out by a triple quadrupole, the signal intensity (CPS) of the characteristic ions was obtained in the detector, and the mass spectrometry file of the sample downstream was opened by MultiQuant software to carry out chromatographic peak integration and correction, where the peak area (area) of each chromatographic peak represented the relative content of the corresponding substance [78].

#### 4.2.5. Differential Metabolites (DMs) Selected

The measurement of plant hormones was accomplished using liquid chromatography–tandem mass spectrometry (LC-MS/MS), following the experimental procedures established by previous studies [35]. Detection of phytohormone expression data was performed by Hangzhou Jingjie Bio-technology Co.

For two-group analysis, DMs were determined by VIP (VIP > 1) and absolute Log2FC (|Log2FC| ≥ 1.0). VIP values were extracted from OPLS-DA results generated using R package MetaboAnalystR. The data were log-transformed (lo$g_{2}$) and mean-centered before OPLS-DA. In order to avoid overfitting, a permutation test (200 permutations) was performed.

#### 4.2.6. OPLS-DA and Pearson Correlation Coefficients

The supervised OPLS-DA model was analyzed using the MetaboAnalystR package OPLSR and the Anal function in R (1.0.1) (www.r-project.org (accessed on 1 November 2023)). The Pearson correlation coefficients (PCCs) between the samples were then calculated using the cor function in R and presented as heatmaps. The PCC analysis was performed using the R package ComplexHeatmap (0.84).

### 4.3. Determination of Chlorophyll Fluorescence Parameters

The assay for the determination of chlorophyll fluorescence parameters was based on a previous experimental technique [79]. The PSII chlorophyll fluorescence parameters and related parameters in *R. chrysanthum* leaves were determined and calculated using the IMAGING-PAM chlorophyll fluorescence imaging system (Heinz Walz, Germany). The test materials were kept in darkness for 30 min, after which three observation points on leaves with similar growth were chosen, and the minimal fluorescence (Fo), maximum fluorescence (Fm), maximum fluorescence in the light (Fm’), and photochemical quenching (qL) were measured and the related parameters were calculated, as well as the fast light response curves of the non-photochemical quenching (NPQ) and the actual electron transport rate (ETR).

## Data Availability

The data used in this study are available from the corresponding author on submission of a reasonable request. The data are not publicly available due to privacy.

## Supplementary Materials

The following supporting information can be downloaded at: https://www.mdpi.com/article/10.3390/plants13162232/s1, Table S1: The analysis of metabolite profiles of *R. chrysanthum* leaves; Table S2: Information on the differential metabolites of comparison groups M and N; Table S3: Information on the differential metabolites of comparison groups N and Q.

## Abbreviations

ABA	Abscisic acid
ANOVA	Analysis of variance
DMs	Differential metabolites
ETR	Actual electron transport rate
FC	Fold change
Fm	Maximum fluorescence
Fm’	Maximum fluorescence in the light
Fo	Minimal fluorescence
Fv/Fm	Maximal photochemical efficiency of PSII
JA	Jasmonic acid
JA-L-Ile	Jasmonoyl-L-isoleucine
JA-Val	Jasmonoyl-L-valine
KEGG	Kyoto Encyclopedia of Genes and Genomes
MWDB	Metware database
NPQ	Non-photochemical quenching
OPLS-DA	Orthogonal partial least-squares discriminant analysis
PAR	Photosynthetic active radiation
PCCs	Pearson’s correlation coefficients
PSII	Photosystem II
QCs	Quality control samples
*R. chrysanthum*	*Rhododendron chrysanthum* Pall.
SD	Standard deviation
UPLC-MS/MS	Ultra-performance liquid chromatography–tandem mass spectrometry
UV-B	Ultraviolet radiation b
VIP	Variable importance in projection
Y(II)	Photochemical yield of PSII
